# Metabolic features of enantiornithines (Avialae: Ornithothoraces): evidence supporting a semi-precocial/precocial developmental trajectory

**DOI:** 10.1098/rsos.260574

**Published:** 2026-10-21

**Authors:** Mariana V. Haase, Fabien Knoll, Dennis Voeten, Sophie Sanchez, Zhiheng Li, Hugo Bert, Esaú V. de Araújo, Francisco J. Serrano, Javier C. Terol, Jesús Marugán-Lobón, Jorge Cubo

**Affiliations:** ^1^ Sorbonne Université, Muséum national d'Histoire naturelle, CNRS, Centre de Recherche en Paléontologie—Paris (CR2P, UMR 7207), 4 Place Jussieu, 75005 Paris, Île-de-France, France; ^2^ Morton K. Blaustein Department of Earth & Planetary Sciences, Johns Hopkins University, Baltimore, ML, USA; ^3^ Departamento de Paleobiología, Museo Nacional de Ciencias Naturales—CSIC, Madrid, Spain; ^4^ Frisian Museum of Natural History, Leeuwarden, The Netherlands; ^5^ European Synchrotron Radiation Facility, Grenoble, Auvergne-Rhône-Alpes, France; ^6^ Department of Organismal Biology, Subdepartment of Evolution and Development, Uppsala University, Uppsala, Uppsala County, Sweden; ^7^ Key Laboratory of Vertebrate Evolution and Human Origins, Institute of Vertebrate Paleontology and Paleoanthropology, Chinese Academy of Sciences, Beijing, People's Republic of China; ^8^ Université Lyon 1, ENS de Lyon, Université Jean Monnet, CNRS, LGL TPE, UMR 5276, Villeurbanne, Auvergne-Rhône-Alpes, France; ^9^ Laboratório de Sistemática e Tafonomia de Vertebrados Fósseis (LAPUG), Departamento de Geologia e Paleontologia, Museu Nacional, Universidade Federal do Rio de Janeiro, Brazil; ^10^ Dinosaur Institute, Natural History Museum of Los Angeles County, Los Angeles, CA, USA; ^11^ Departamento de Ecología y Geología, Facultad de Ciencias, Universidad de Málaga, Málaga, Spain; ^12^ Unidad de Paleontología, Dpto. Biología, Universidad Autónoma de Madrid, Madrid, Spain

**Keywords:** developmental strategy, osteohistology, paleophysiology, phylogenetic methods

## Abstract

The enantiornithes, a group of Mesozoic birds, have morphological features suggesting a precocial mode of development; however, their detailed physiological characteristics, particularly hatchling metabolic rates, have yet to be explored. Here, we retrodicted metabolic rates for four Lower Cretaceous enantiornithines: one hatchling, one adult from Las Hoyas, one early post-hatching juvenile and one post-juvenile individual from the Jehol Biota, using phylogenetic comparative analyses and a comprehensive dataset of extant birds spanning the altricial–precocial spectrum. By examining vascular density (related to resting metabolic rate (RMR)) and nutrient foraminal size (proportional to blood flow rate) as proxies, we inferred the metabolic scope of the hatchling enantiornithine and determined that it fell toward the precocial end of the altricial–precocial spectrum. The RMR values of the two enantiornithine chicks were retrodicted as 0.67 mlO_2_ h^–1^ g^–0.93^. The peak metabolic rate (PMR) retrodicted is twice the resting metabolism for the Las Hoyas hatchling. These results are consistent with a semi-precocial/precocial development characterized by independent locomotion by climbing using the wing claws soon after hatching. However, flapping flight may have been delayed, owing to incomplete ossification of the sternum, as observed in one hatchling specimen.

## 1. Introduction

Enantiornithes are small to medium-sized avialans from the Cretaceous period, which were globally distributed, as shown by their fossils recovered from different countries including Australia [1], Brazil [2], the USA [3], China [4], Myanmar [5], Argentina [6], Madagascar [7] and Spain [8–10]. There is strong evidence that enantiornithines were primarily arboreal [11,12]. Embryos and hatchlings of this group have been documented in Europe, Asia and South America, and their precocial mode of development is evidenced by hatchling specimens with well-ossified skeletons, large egg sizes and feathers [13–19].

The developmental modes of extant birds are categorized based on the condition of the chick at hatching, which varies significantly among species. Altricial hatchlings, such as passerines, hatch in an early developmental state and rely heavily on parental care, whereas precocial hatchlings are more advanced at hatching, enabling greater functional independence [20,21]. Semi-precocial (e.g. Procellariidae) and precocial (e.g. Phasianidae) nestlings are characterized by open eyes at hatching, a well-developed downy plumage, advanced motor capabilities and the ability to locomote independently shortly after emergence from the egg [22,23]. These developmental modes differ primarily in the degree of postnatal parental provisioning: semi-precocial nestlings rely on parental feeding, whereas precocial hatchlings are capable of self-feeding or may follow their parents to foraging sites within hours of hatching [20–25]. Superprecocial species, such as Megapodiidae, can fly or run shortly after hatching, once their feathers are dry [22]. By contrast, the hatchlings of altricial species, such as Passeriformes and Psittaciformes, hatch naked, with closed eyes- and are unable to locomote or autonomously regulate their body temperature [24].

Altricial and precocial avialan embryos also differ notably in their patterns of oxygen consumption (VO₂) during incubation. In altricial species, oxygen consumption increases continuously throughout embryonic development, whereas in precocial species it rises during early and mid-incubation but reaches a plateau during late incubation, resulting in stabilization of VO₂ and a shift towards tissue maturation [24]. Hatched chicks, regardless of developmental mode, typically have lower metabolic rates than those of adults of the same species. Postnatal metabolism in birds is primarily driven by body mass (BM) through allometric scaling and ontogeny acting through changes in body size [25–27].

As development progresses, metabolic capacity increases, as reflected in a rise in resting metabolic rate (RMR) at thermoneutrality [28–31]. In precocial species, the adult RMR coincides with the achievement of thermoneutrality and in altricial ones the adult RMR is reached at the end of the growth period [20,32]. After hatching, peak metabolic rates (PMRs) (i.e. maximum mean of oxygen consumption rate VO_2_ observed during exposure to sub-thermoneutral temperatures) of precocial chicks increase with BM, whereas the PMR is initially equal to the RMR in altricial chicks and only later, with subsequent growth, do their PMR increase in response to the cold stress [20,32,33].

Visser [20] reviewed thermoregulation in neonatal birds and found that altricial hatchlings exhibit similar RMR and PMR values, while precocial hatchlings have much higher PMR/RMR ratio values. For example, in superprecocial species, such as the megapode malleefowl (*Leipoa ocellata*) hatchling, the PMR is more than four times the RMR value. This distinction arises from differences in sensory and motor capabilities between altricial and precocial chicks; for example, megapode chicks emerge from mounds with no parental care and begin foraging immediately after hatching [34].

In this study, we retrodicted the RMR and PMR values of two fossil hatchlings of enantiornithines: a hatchling from the Las Hoyas site, MCCM-LH-26189, and the early immature IVPP V15686 from Jehol Biota. We inferred and compared their metabolic profiles with those of extant bird species using measurements from a comprehensive list of resting and PMRs of hatchlings [20]. We then incorporated a phylogenetic framework using phylogenetic eigenvector maps (PEMs), a method that accounts for evolutionary non-independence while retrodicting metabolic rates. We also revisited the RMR of subadult and adult fossil enantiornithines, *Iberomesornis* sp. MCCM-LH09681 and *Concornis lacustris* MCCM-LH21006. In addition, we estimated the PMR of the post-juveniles *Eoalulavis hoyasi* MCCM-LH-13500 and enantiornithine indet. IVPP V17968. These analyses aimed to better understand the aerobic capacity of enantiornithines and elucidate their metabolic changes throughout ontogeny. Our study contributes to understanding enantiornithines' physiology and offers a tool for exploring the developmental trajectories of fossil avialan hatchlings.

## 2. Materials and methods

### 2.1. Materials

Enantiornithine MPCM-LH-26189 is an almost complete, largely articulated hatchling skeleton [8]. The enantiornithine MCCM-LH09681 (*Iberomesornis* sp.) was considered a subadult whereas MCCM-LH21006 (*Concornis lacustris*) and MCCM-LH-13500 (*Eoalulavis hoyasi*) were previously interpreted to represent adult specimens [9,11,35,36] (supplementary material, spreadsheet S1). These specimens originate from the Early Cretaceous wetland deposits of the Las Hoyas fossil site [8,37] and are currently housed at the Museo de Paleontología de Castilla-La Mancha, Cuenca, Spain. Chinese enantiornithine specimens represent an early post-hatching (IVPP V17968), evidenced by an only partially ossified sternum, and a subadult (IVPP V15686) [38], which preserves apparent fusion of the hindlimb elements but no evident sternal fusion. These individuals originate from the Jiufotang Formation (120 Ma) in the Jehol Biota [38] and are currently housed at the Institute of Vertebrate Paleontology and Paleoanthropology of the Chinese Academy of Sciences in Beijing, China (IVPP). Based on its highly vascularized woven bone matrix, IVPP V15686 was identified as a very young post-hatching individual by O'Connor *et al.* [38] and is considered a hatchling in the present analysis. BM (in grams) of *Iberomesornis*, *Concornis* and *Eoalulavis* was obtained based on the works of Serrano *et al.* [9,39]. For the other fossil specimens, the BM was estimated using the width at femoral midshaft (dFW; in mm), applying equation (2.1) described by Serrano *et al.* [39]:

(2.1) $$\begin{array}{r} \log BM=-1.785+2.330\;\log(d_{\mathrm{FW}}). \end{array}$$

### 2.2. Resting and peak metabolic rate retrodiction

For the comparative analyses, the two immature specimens (MPCM-LH-26189 and IVPP V15686) were considered to represent the same ontogenetic stage and their type of development was retrodicted based on metabolic rate using data from a published source (extant data, table 5.4) [20]. To generate our dataset, we compiled previously published data and included our own measurements of neonatal metabolic rates (RMR and PMR) of bird species representing the full range of developmental modes along the altricial–precocial spectrum. Categories and features for the development type in hatchlings came from Nice [23] and Starck & Ricklefs [40]. The BM, RMR and PMR (in Watts; W) values for hatchlings of extant species were obtained primarily from Visser [20]. The mass-specific (i.e. metabolic rate by BM) RMR and PMR (mlO_2_ h^–1^ g^–1^) were calculated as the rate at which birds consume energy per gram of body weight, using the conversion equation from Vleck *et al.* [41], where 1.16 mW = 0.21 mlO_2_ h^–1^. Modern data from hatchlings of one turtle and three crocodylian species and metabolic data of three adult crocodylians served as the outgroup for our analyses. Mass-specific metabolic rates were used only for comparisons of metabolic scope ratios with extant hatchling birds. Retrodictions of RMR and PMR were instead performed using mass-independent metabolic rate scaling applying BM exponents derived from ancestral character estimation across the amniote phylogenetic tree (see §2.3 for details).

We inferred the mass-independent RMR for the chicks using the tibiotarsal vascular density, which was predicted using ordinary least-squares linear regression implemented in R version 4.4.1 [42]. The regression model was fitted to log-transformed femoral and tibiotarsal vascular density data from species with complete measurements, and predicted values were back-transformed to the original scale using the exponential function. This model was applied to estimate vascular density in nine specimens (*p* = 0.000235; *R*^2^ = 0.94; see supplementary materials, spreadsheet S2).

Measured femoral and tibiotarsal vascular densities, along with estimated tibiotarsal vascular densities, are provided in the spreadsheets S2 and S3 in the supplementary material. To retrodict the RMR values of both hatchlings, an inference model was developed using data from 17 hatchlings of extant bird species that were primarily determined in previous studies (supplementary material, spreadsheet S4). In addition, histological data from a hatchling of *Ficedula albicollis* available in the virtual histology collection of the Department of Organismal Biology, Uppsala University, Sweden, were included (table 1). The vascular density, which was quantified by dividing the number of vascular canals by total bone area (number of vascular canals µm²) [43,44] using ImageJ/Fiji [45], served as the explanatory variable, while metabolic rate data were derived from McFarlane *et al.* [46]. The tibiotarsal vascular density was quantified based on micro-computed tomography (µ-CT) images using propagation phase-contrast synchrotron µ-CT at the European Synchrotron Radiation Facility, Grenoble, France (ESRF) for MPCM-LH-26189 on beamline ID19 (supplementary material, fig. S1), real cross-sections from the collections of the IVPP in the enantiornithine specimens IVPP V15686 (supplementary material, fig. S2), and from available published data for *Dromaius novaehollandiae*, *Phasianus colchicus*, *Alectoris rufa*, *Turdus merula*, *Anas platyrhynchos*, *Struthio camelus*, *Aptenodytes patagonicus*, *Meleagris gallopavo* and *Coturnix japonica* (supplementary material, spreadsheet S2)*.* Tibiotarsal vascular density was estimated using linear regression based on the femur vascular density of seven extant species: *Ficedula albicollis*, *Callipepla californica*, *Larus occidentalis*, *Falco sparverius*, *Pyrrhura molinae*, *Aphelocoma californica* and *Haemorhous mexicanus* (supplementary material, spreadsheet S3).

**Table 1.** List of recent and fossil museum specimens sampled for each metabolic rate metric.

taxon	ontogenetic state	collection number	vascular density(vascular canals µm²)	*Q̇* (ml s^−1^)	metabolic rate metric
enantiornithes indet. †	hatchling	MPCM-LH-26189	0.0000651	0.0000144	RMR and PMR
enantiornithes indet. †	hatchling	IVPP V15686	0.0000909		RMR
*Concornis lacustris* †*	adult	MCCM-LH21006	0.0039		RMR
*Iberomesornis* sp. †*	subadult	MCCM-LH09681	0.00331		RMR
enantiornithes indet. †	subadult	IVPP V17968		0.0009031	PMR
*Eoalulavis hoyasi* †	adult	MCCM-LH-13500		0.0000606	PMR
*Bubulcus ibis*	hatchling	MNCN 27070		0.0001585	PMR
*Turdus merula*	hatchling	MNHN ZO 2020		0.0005801	PMR
*Sterna hirundo*	hatchling	MNHN ZO 2010 640		0.0000322	PMR
*Sterna fuscata*	hatchling	MNHN ZO AC 1996 2		0.0000885	PMR
*Larus ridibundus*	hatchling	MNHN ZO 1997-959		0.0008986	PMR
*Rissa tridactyla*	hatchling	MNHN ZO AC 1997 1114		0.0001585	PMR
*Oceanodroma leucorhoa*	hatchling	MNHN ZO AC 2001 316		0.0000322	PMR
*Gallus domesticus*	hatchling	MNHN ZO AC 1885 2109		0.0001409	PMR
*Meleagris gallopavo*	hatchling	MNHN ZO 1990 50		0.0002720	PMR
*Charadrius dubious*	hatchling	MNCN 23126-02		0.0012149	PMR
*Calidris alpina*	hatchling	MNHN ZO AC 2000 291		0.0000178	PMR
*Phalacrocorax aristotelis*	hatchling	MNHN ZO AC 1996 5		0.0081709	PMR
*Anas crecca*	adult	MNCN 23608		0.0006899	PMR
*Columba rupestris*	adult	UWBM 59986		0.0002837	PMR
*Coturnix ceca*	adult	MAUV-711		0.0001692	PMR
*Eudyptula minor*	adult	UWBM 80409		0.0004821	PMR
*Haliaeetus leucocephalus*	adult	UWBM 33937		0.0079373	PMR
*Larus argentatus*	adult	UWBM 56577		0.0016506	PMR
*Phalacrocorax carbo*	adult	UWBM 59908		0.0016118	PMR
*Tyrannus tyrannus*	adult	UWBM 35764		0.0000164	PMR
*Sturnus unicolor*	adult	MNCN 26971		0.0000164	PMR
*Corvus corax*	adult	UWBM 59566		0.0053022	PMR
*Pelecanus erythrorhynchus*	adult	UWBM 59227		0.0030430	PMR
*Falco peregrinus*	adult	UWBM 78840		0.0006336	PMR
*Rhea americana*	adult	LACNHM 93063		0.0027661	PMR

*Measurements extracted from Cubo *et al.* [11].

To retrodict the PMR values (i.e. the highest rate of oxygen consumption during arduous activity) of the enantiornithine hatchling MPCM-LH-26189, a PEM model using $\dot{Q}$ as a co-predictor was constructed using data from 67 hatchlings of extant species. Twelve of these are housed in the Museo Nacional de Ciencias Naturales de Madrid (MNCN-CSIC)-Madrid, the ESRF-Grenoble, and the Muséum National d'Histoire Naturelle-Paris (MNHN) (table 1). Their nutrient foramina apertures were measured from three-dimensional (3D) reconstructions of femora and tibiotarsi carried out in VG Studio MAX® and Mimics®. For comparative purposes, the foramen aperture area was approximated as a geometric circle, and the minor diameter of the circle was used to estimate the nutrient artery blood flow rate ($\dot{Q}$; ml s^–1^) following the method of Seymour *et al.* [47]. This approach infers regional blood flow by quantifying the cross-sectional aperture area of vascular foramina in long bones. A nutrient foramen was identified in the femur of the MPCM-LH-26189 using the synchroton µ-CT at the FaXToR beamline (BL31) of the ALBA synchrotron. The value of $\dot{Q}$ was subsequently estimated from the diameter of the nutrient foramen aperture (see supplementary material, fig. S3, spreadsheet S1 for measurement details). For extant taxa lacking neonatal representatives in the surveyed museum collections, the blood flow rates were estimated using a regression-based predictive equation (2.2) provided by Hu *et al.* [48]. Those estimations allowed us to examine the scaling relationships of long bone blood flow rates on BMs (see supplementary material, spreadsheet S4 for details).

(2.2) $$\begin{array}{r} \dot{Q}=3.6\times 10^{-5\;}BM^{1.22\pm 0.84}. \end{array}$$

The metabolic scope values of the hatchling MPCM-LH-26189 were calculated as the ratio between its mass-specific PMR and RMR values and compared with those of living hatchlings of extant species. To retrodict the PMR of the mature individuals, a model was constructed using the PMR data from adult individuals of bird species and their femoral $\dot{Q}$ values as reported by Allan *et al.* [49] for IVPP V17968 and the measured humeral $\dot{Q}$ values of 13 mature bird specimens of the museum collections for *E. hoyasi*, MCCM-LH-13500 (supplementary material, spreadsheets S5 and S6).

$\dot{Q}$ was calculated based on the femoral foramen radius in the mature individual (IVPP V17968) and on the humeral foramen radius in *E. hoyasi* (supplementary material, figs. S4 and S5), measured using ImageJ/Fiji from µ-CT images [45]. Estimates of $\dot{Q}$were made using the following polynomial equation (2.3) [47]:

(2.3) $$\begin{array}{r} log\dot{Q}=-0.20\;log\;{r_{i}}^{2}+1.91\;log\;r_{i}+1.82, \end{array}$$

where r_i_ is the arterial lumen radius extracted from the arterial lumen area, which roughly corresponds to 20% of the total foramen area [50].

### 2.3. Phylogenetic framework

The phylogenetic relationships of Neornithes were derived from Cooney *et al.* [51]. Crawford *et al.* [52] and Brusatte *et al.* [53] provided the Archelosauria and Archosauria outgroup topology, respectively.

To reduce the influence of BM across sauropsids, we first retrodicted the mass-independent metabolic rate, rather than the mass-specific metabolic rate. To calculate mass-independent metabolic rates BM scaling exponents were obtained using the method described by Sena *et al.* [54] within a phylogenetic framework constructed in Mesquite [55]. For the extant amniotes, mass-independent metabolic rates were calculated by assigning BM scaling exponents of 0.829 to turtles and crocodylian species [56] and 1.02 to avialans [49]. The phylogenetic mean scaling exponent was applied to correct metabolic rates for BM effects, thereby producing mass-independent metabolic rates that represent variation beyond allometric scaling. This procedure yielded mass-independent RMR and PMR units of mlO_2_ h^–1^ g^–0.93^ and mlO_2_ h^–1^ g^–0.92^, respectively.

The mass-specific RMR and PMR from retrodicted mass-independent values were calculated using equations (2.4) and (2.5):

(2.4) $$Mass-specific\;RMR=Mass-independent\;RMR\times BM^{\left(mass-independent\;RMR\;exponent-1\right)}$$

and

(2.5) $$Mass-specific\;PMR=Mass-independent\;PMR\times BM^{\left(mass-independent\;PMR\;exponent-1\right)}.$$

### 2.4. Statistical and phylogenetic comparative methods

Tibiotarsal vascular density in extant hatchlings with missing data was estimated using a linear regression model that inferred the relationship between femoral and tibiotarsal vascular densities, using the package ‘dplyr’ version 1.1.4 [57] in R version 4.4.1 [42]. Paleobiological inference models adapted from the R script reported by Legendre *et al.* [44] and Bert *et al.* [58] for mass-independent RMR and PMR were constructed using PEMs from the ‘MPSEM’ package version 0.5.1 [59] in R [42]; these models allowed retrodiction of the RMR and PMR values, along with their 95% confidence intervals (CIs). The normality of residuals was checked using the Shapiro–Wilk test in R [42].

To assess differences in body size distributions between enantiornithines from the Jehol Biota (*n* = 45) and those reported from Spain (*n* = 4), we compiled BM estimates from the literature [39,60]. Analyses were conducted in R version 4.4.1 [42] using the packages ‘ggplot2’ version 4.0.3 [61], ‘rstatix’ version 0.7.3 [62] and ‘coin’ version 1.4-3 [63]. Differences between groups were evaluated using a Wilcoxon rank-sum test [64] in R [42].

Finally, differences in metabolic scope among developmental modes were assessed using a permutation-based linear model implemented in the R package ‘RRPP’ version 2.1.2 [65]. Type of development was treated as a predictor and PMR/RMR ratio as the response variable. Pairwise comparisons among developmental modes were performed using least-squares mean and permutation-based tests. Then, the developmental mode of our fossil hatchling MPCM-LH-26189 was estimated based on its PMR/RMR value.

## 3. Results

Hatchling MPCM-LH-26189 from Las Hoyas and the young post-hatchling IVPP V15686 from the Jehol Biota yielded the same retrodicted mass-independent RMR values, with a value of 0.67 mlO_2_ h^–1^ g^–0.93^ (with 95% CI = 0.39–1.02), based on phylogenetic eigenvectors 1, 2, 3, 4, 5, 11, 12 and 18, and the tibiotarsal vascular density inference model (*R*^2^ = 0.97; Akaike information criterion corrected (AICc) = 21.55; *p* = 1.07 × 10^–7^) (figure 1).

**Figure 1.**
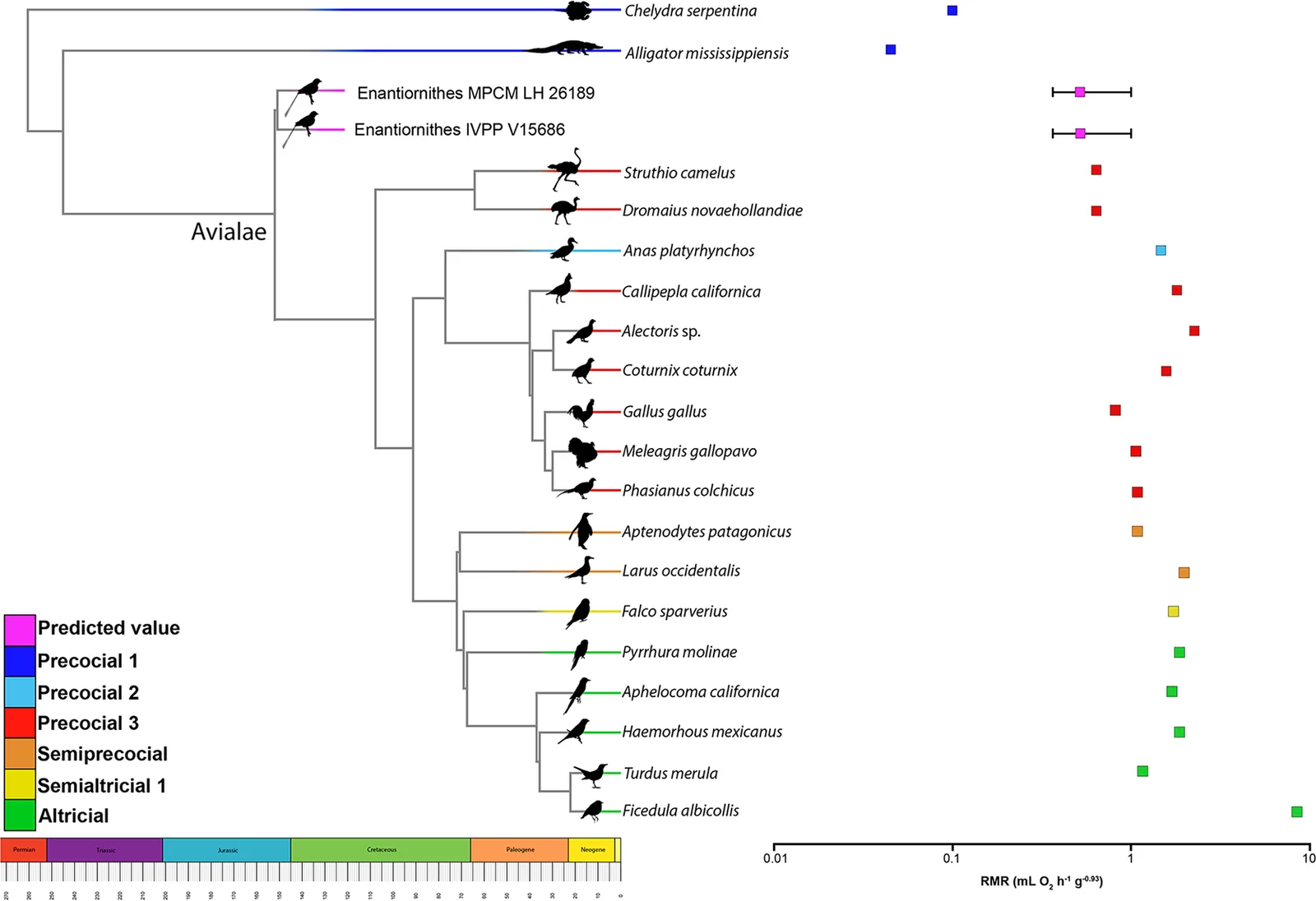
Observed and retrodicted mass-independent resting metabolic rate (RMR) values of extant hatchlings and early post-hatching fossil chicks, MPCM-LH-26189 and IVPP V15686. The RMR was calculated using tibiotarsal vascular density as a co-predictor in phylogenetic eigenvector map (PEM) analysis (lilac squares). The RMR values for hatchlings of extant bird species are plotted in various colours, each representing a distinct mode of development: *altricial* (green) chicks hatch featherless and are entirely dependent on parental care, *semi-altricial 1* (yellow) chicks remain in the nest but are born with some feathering, *semi-precocial* (orange) chicks show early locomotor activity but remain reliant on parental feeding, *precocial 3* (light red) chicks can eat by themselves, and *precocial 2* (sky blue) chicks can forage independently, but continue to receive parental care. Branches are proportional to time. The archelosaur silhouettes have been modified and adapted from copyright-free images provided by Phylopic (http://www.phylopic.org).

The comparative analysis shows that the early juvenile Asian enantiornithine had a larger estimated BM (27.85 g ± 7.2) than the young European specimen (2.09 g ± 0.5). BM estimates for additional enantiornithine individuals across ontogenetic stages are provided in spreadsheet S1 in the supplementary material.

Mass-specific RMR of 0.53 (0.31–0.81) mlO₂ h⁻¹ g⁻¹ and 0.64 (0.37–0.97) mlO₂ h⁻¹ g⁻¹ were obtained for IVPP V15686 (BM: 27.85 g) and MPCM-LH-26189 (BM: 2.09 g), respectively (table 2).

**Table 2.** Mass-specific metabolic rate values retrodicted for the sampled fossil enantiornithines.

taxon	ontogenetic state	collection number	RMR(ml O₂ h⁻¹ g⁻¹)	PMR(ml O₂ h⁻¹ g⁻¹)	PMR/RMR
enantiornithes indet. †	hatchling	MPCM-LH-26189	0.64 (0.37 – 0.97)	1.52 (1.23 – 1.87)	2.38 (1.93 – 3.33)
enantiornithes indet. †	hatchling	IVPP V15686	0.53 (0.31 – 0.81)		
*Concornis lacustris* †	adult	MCCM-LH21006	1.49 (1.36 – 1.62)		
*Iberomesornis* sp. †	subadult	MCCM-LH09681	2.34 (2.23 – 2.57)		
enantiornithes indet. †	subadult	IVPP V17968		4.90 (3.80 – 6.30)	
*Eoalulavis hoyasi* †	adult	MCCM-LH-13500		7.70 (4.10 – 14.44)	

The mass-independent PMR value for the hatchling MPCM-LH-26189 was 1.61 mlO_2_ h^–1^ g^–0.92^ (with 95% CI = 1.31–1.98), using PEMs 1, 2, 3, 4, 6, 7, 10, 11, 14, 15, 17, 19, 21, 22, 23, 24, 25, 26, 27, 28, 32, 33, 34, 35, 38, 39, 41, 47, 56, 60, 65 and 66, and estimated $\dot{Q}$ value as the co-predictor (*R*^2^ = 0.97; AICc = 23.86; *p* < 2.2 × 10⁻^16^) (figure 2). Leave-one-out cross-validation showed no significant difference between predicted and observed values for extant species (*p* = 0.16 in RMR analysis and *p* = 0.61 in PMR analysis).

**Figure 2.**
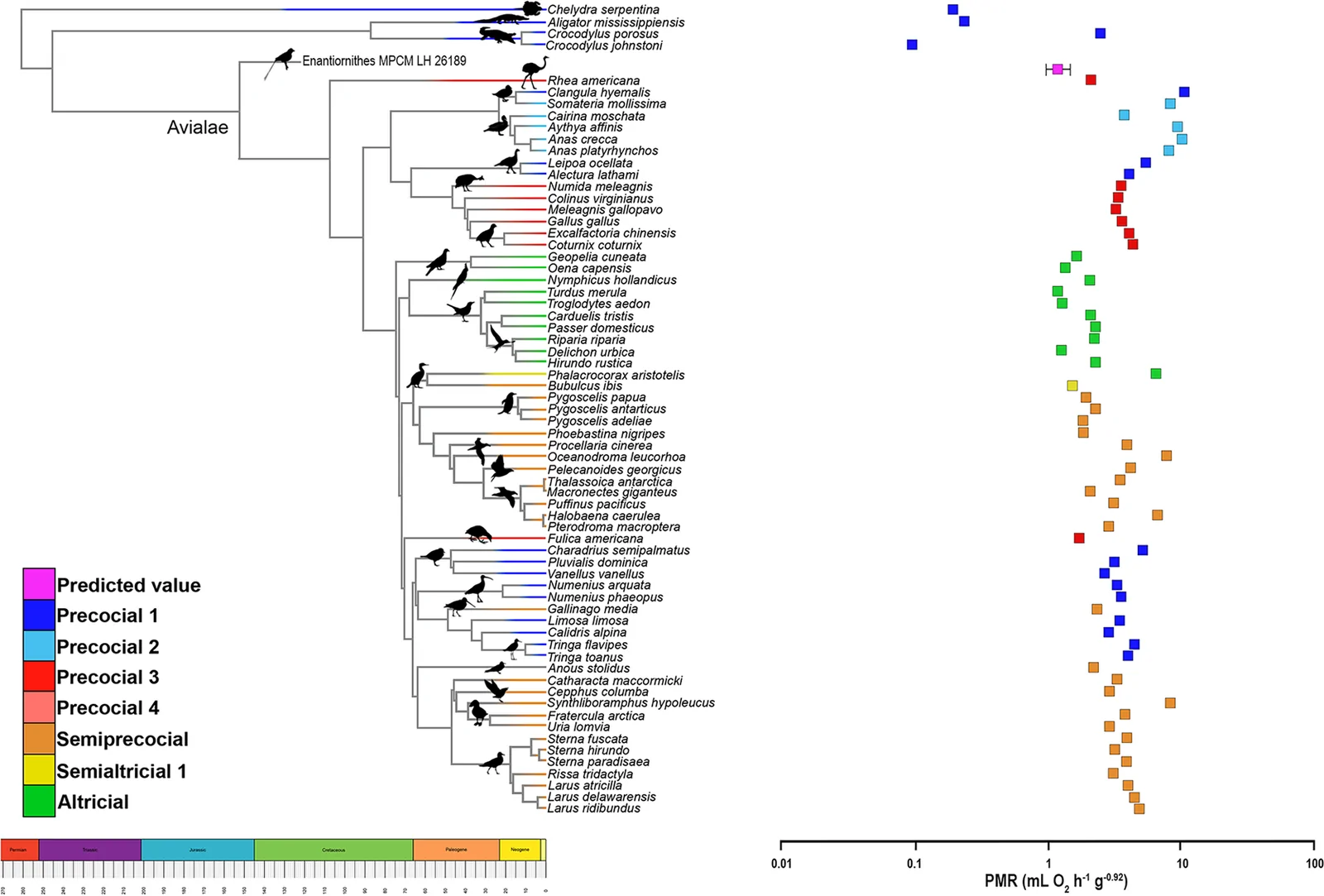
Observed and retrodicted mass-independent peak metabolic rate (PMR) values for extant bird species and for the hatchling MPCM-LH-26189. The retrodicted PMR value was estimated using femoral nutrient foramen area and derived blood flow ($\dot{Q}$; ml s⁻¹) as a co-predictor in the phylogenetic eigenvector map (PEM) analysis (indicated by lilac square). PMR values for hatchlings of extant bird species are displayed with corresponding colours, representing their different developmental trajectories: *altricial* (green) chicks hatch featherless and are entirely dependent on parental care; *semi-altricial 1* (yellow) chicks remain in the nest but are born with some feathering; *semi-precocial* (orange) chicks show early locomotor activity, but remain reliant on parental feeding; *precocial 4* (coral) chicks follow their parents post-hatching; *precocial 3* (light red) chicks can eat by themselves; *precocial 2* (sky blue) chicks can forage independently but continue to receive parental care; *precocial 1* (vivid blue) chicks have no interactions with parents. Branches are proportional to time. The archelosaur silhouettes have been modified and adapted from copyright-free images provided by Phylopic (http://www.phylopic.org).

Based on BM, we estimated mass-specific PMR value of 1.52 (1.23–1.87) mlO₂ h⁻¹ g⁻¹ for MPCM-LH-26189. The factorial metabolic scope value (ratio of PMR to RMR) of the Las Hoyas chick was 2.38 (1.93–3.33).

The mass-independent PMR value for the post-juvenile IVPP V17968 was 5.08 mlO_2_ h^–1^ g^–0.95^ (with 95% CI = 4.69–7.87), based on the femoral $\dot{Q}$ value observed as the co-predictor (*R*^2^ = 0.88; AICc = 30.02; *p* = 6.651 × 10^–06^) using PEMs 1, 3, 9 and 11 (figure 3). For *E. hoyasi*, MCCM-LH-13500, the mass-independent PMR was 12.12 mlO_2_ h^–1^ g^–0.86^ (with 95% CI = 6.99–24.61), based on the humeral $\dot{Q}$ value observed as the co-predictor (*R*^2^ = 0.50; AICc = 44.60; *p* = 0.0042) using eigenvector 1 (figure 4). Leave-one-out cross-validation revealed no significant difference between predicted and observed values for extant species (*p* = 0.90 and 0.98, respectively). We calculated mass-specific PMR values of 4.90 (3.80–6.30) mlO₂ h⁻¹ g⁻¹ for IVPP V17968 and 7.70 (4.10–14.44) mlO₂ h⁻¹ g⁻¹ for *Eoalulavis hoyasi* MCCM-LH-13500, accounting for a BM of 96.78 g ± 24.97 estimated in the present study and 45 g ± 9 provided by Serrano *et al.* [9], respectively.

**Figure 3.**
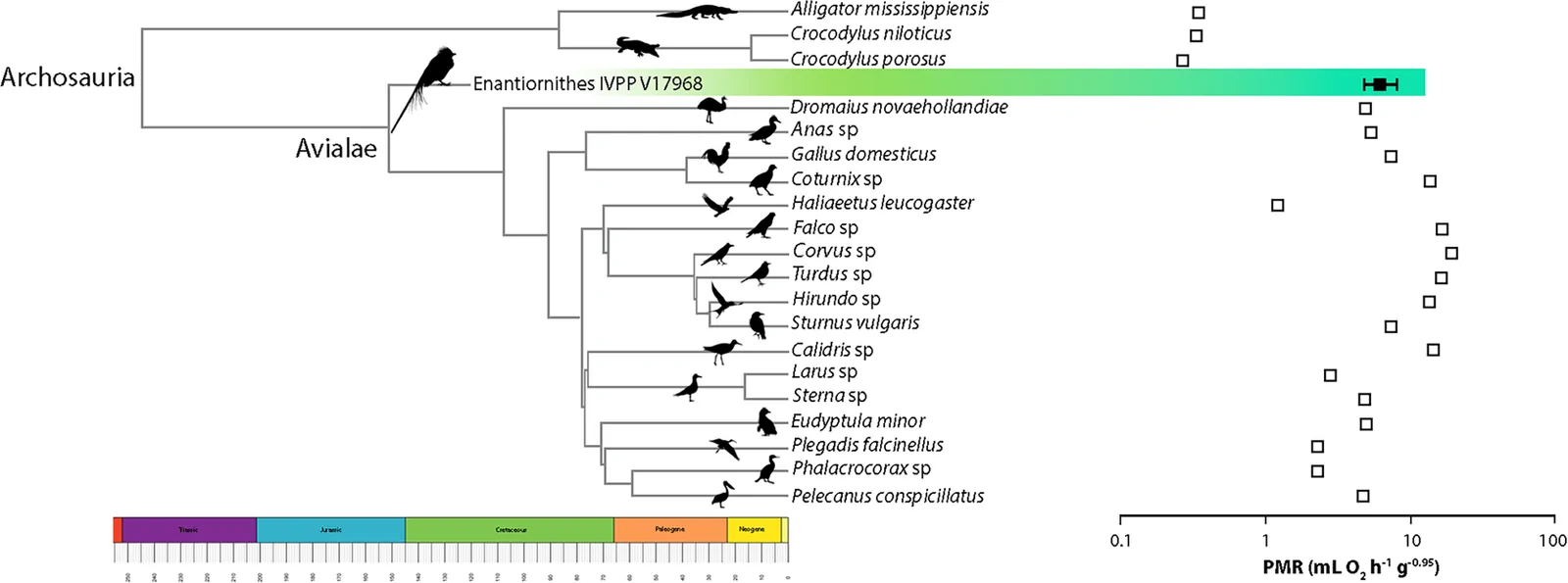
Time-calibrated phylogenetic relationships among extant sauropsid taxa used to construct the mass-independent PMR inference model, together with the extinct mature enantiornithine IVPP V17968. PMR was retrodicted using femoral nutrient foramen area and derived blood flow ($\dot{Q}$) as a co-predictor. Branch lengths are proportional to divergence time. White squares indicate observed PMR values for extant taxa, whereas the black square denotes the retrodicted PMR value for the enantiornithine specimen. Horizontal bars represent the 95% CI of the retrodicted estimate. The archosaur silhouettes have been modified and adapted from copyright-free images provided by Phylopic (http://www.phylopic.org).

**Figure 4.**
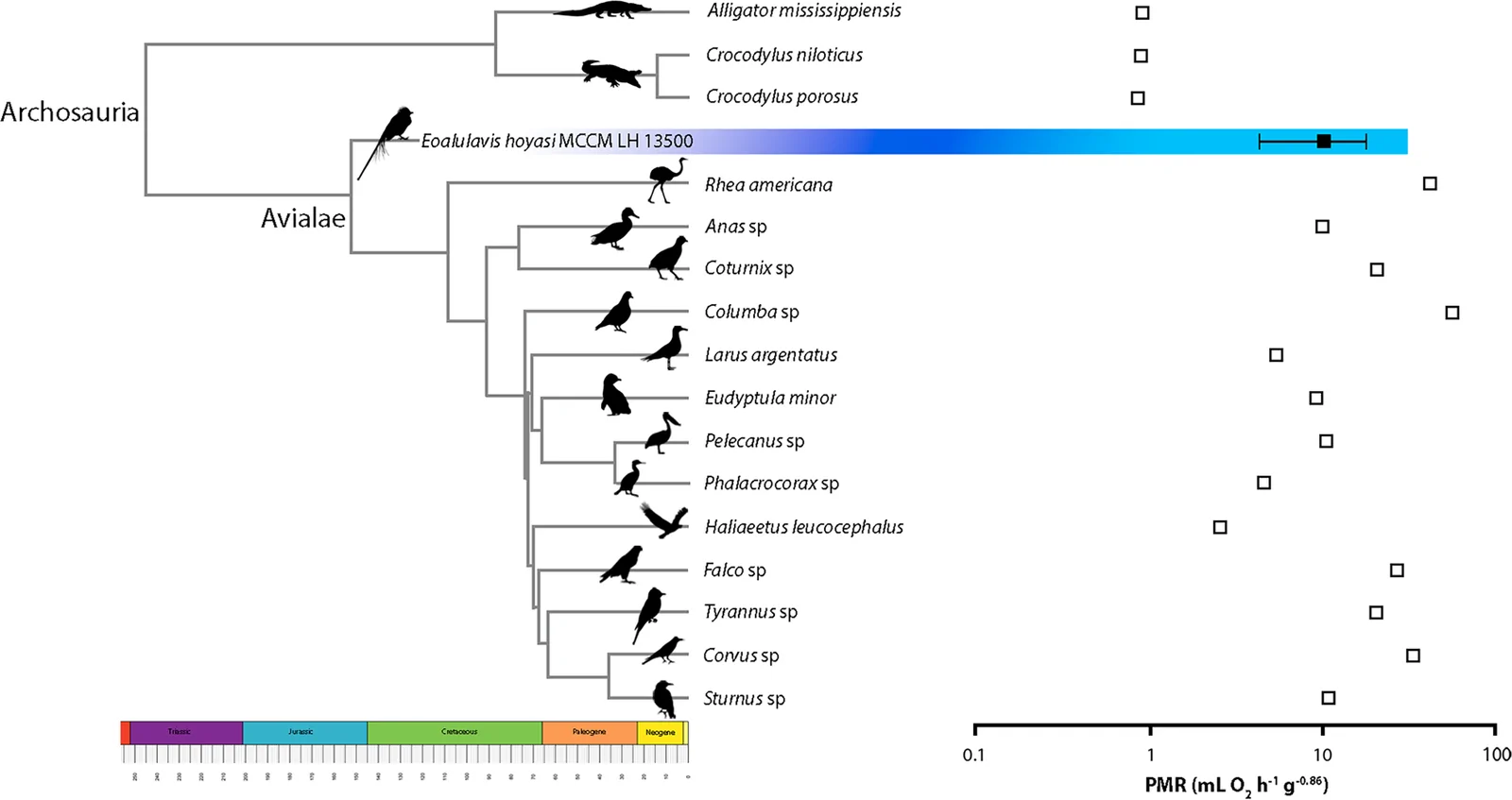
Phylogenetic relationships among extant taxa used to construct the mass-independent maximum metabolic rates inference model and the adult *Eoalulavis hoyasi* MCCM-LH-13500 for which we performed retrodictions using humeral $\dot{Q}$ as a co-predictor. Branches are proportional to time. White squares indicate observed PMR values for extant taxa, whereas the black square represents the retrodicted PMR for *E. hoyasi*; horizontal bars denote the associated 95% CI. The archosaur silhouettes have been modified and adapted from copyright-free images provided by Phylopic (http://www.phylopic.org).

Moreover, we revisited the mass-independent RMR of the post-juvenile individuals of *Concornis* and *Iberomesornis* previously retrodicted by Cubo *et al.* [11]. Accounting for BM estimated at 70 and 20 g, we obtained 1.49 mlO₂ h⁻¹ g⁻¹ and 2.34 mlO₂ h⁻¹ g⁻¹ of mass-specific RMR for *Concornis* (MCCM-LH21006) and *Iberomesornis* (MCCM-LH09681), respectively.

Jehol enantiornithines exhibited a different body size distribution than of Spanish taxa (Wilcoxon rank-sum test: *W* = 164, *p* = 0.003). Jehol Biota taxa span a broader body-size range and include larger-bodied ones, whereas the Spanish birds are concentrated in the small-bodied range. The effect size associated with the difference in body size distribution was moderate (*r* = 0.386), but the sample size unevenness (Jehol Biota: *n* = 45 versus Spain: *n* = 4) requires interpretative caution. Additional specimens from Spain are needed to determine whether the pattern reflects a biogeographic signal rather than sampling bias.

A phylogenetic evaluation with a randomized residual permutation procedure (RRPP) showed an effect of developmental mode on metabolic scope (PMR/RMR) among the sampled extant hatchlings (*F* = 8.13, *R*² = 0.193, *p* = 0.0003). Pairwise comparisons denote that precocial taxa exhibited higher PMR/RMR values than semi-precocial (*d* = 0.964, *p* = 0.0044) and altricial birds (*d* = 1.853, *p* = 0.0003), whereas semi-precocial and altricial taxa did not differ significantly from one another (*d* = 0.889, *p* = 0.0874). The model classified MPCM-LH-26189 as exhibiting a precocial developmental mode, with traits overlapping with the semi-precocial spectrum. The probabilities related to each developmental mode were 0.49 for precociality, 0.44 for semi-precociality and 0.06 for altriciality.

## 4. Discussion

### 4.1. Postnatal growth period and body weight in hatchling enantiornithines

According to our estimates, enantiornithines appear to have experienced rapid growth during the postnatal period, growing to twice hatchling size when reaching the juvenile stage. Compared with previously described enantiornithines, the Las Hoyas specimens tend to be small-bodied, whereas Jehol Biota taxa exhibit a wider range of body sizes [39,60]. For example, the adults of the Spanish enantiornithines species *C. lacustris* and *E*. *hoyasi* weighed approximately 70 and 45 g [9], respectively, whereas subadult individual IVPP V17968 was estimated here to weigh around 96.78 g (as inferred from femoral midshaft width). Furthermore, adult specimens from Las Hoyas were 22–35 times larger than their hatchling counterpart, with fourfold higher estimated $\dot{Q}$ values compared with the hatchling (supplementary material, spreadsheet S1). BM increases during development probably correlate with a transition from a rapid (in early ontogeny) to slower (when approaching maturity) bone growth rate [11,66]. We compared the metabolic rates of the hatchling MPCM-LH-26189 with adult specimens of *C. lacustris* and *E. hoyasi*, and to minimize potential taphonomic and provenance-related effects, ontogenetic metabolic comparisons were conducted separately using hatchling and adult individuals from the same geographic locality (figure 5).

**Figure 5.**
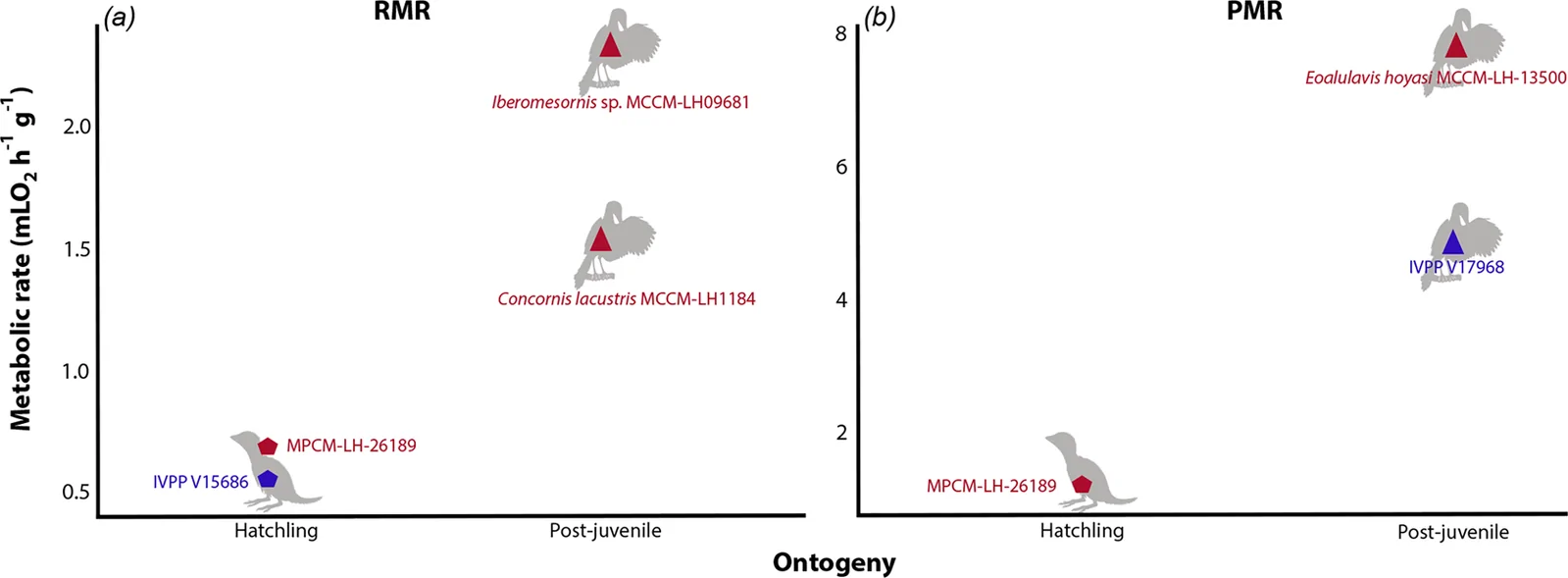
Resting and PMRs across ontogeny in enantiornithines. Mass-specific metabolic rate is shown for chicks, subadults and adult individuals. (*a*) Resting (RMR) and (*b*) peak metabolic rates (PMR). Pentagons indicate hatchlings and triangles indicate the post-juveniles (subadults and adults). Red colour symbols correspond to Spanish avialans, whereas blue colour symbols correspond to Chinese specimens.

### 4.2. Altricial–precocial spectrum mode of development

In enantiornithes, egg and pelvis shapes [18,21,67], advanced limb bone ossification, the presence of feathers and proportionally large brains in known embryos [19,68,69] are all consistent with a restricted precocial developmental mode. This is considered the ancestral avialan condition [70], though some authors argue enantiornithes may even have been superprecocial [71]. The developmental mode inferred from the data for the studied chick specimen indicates self-sufficiency, falling between semi-precocity and precocity, a condition that is observed in, for example, extant Procellariiformes and Charadriiformes [24,72]. RMR increases two- to threefold from early to late ontogeny (figure 1; table 2). The reconstructed metabolic profile of adult enantiornithines from Las Hoyas, based on PMR retrodictions for *E. hoyasi* and RMR estimates for *Concornis,* suggests that these taxa attained maximal metabolic rates approximately five times higher than their RMR (figures 4 and 5). Furthermore, PMR estimates indicate that the small-bodied *E. hoyasi* could have achieved a greater adult mass-specific PMR than the large enantiornithine from the Jehol Biota (figures 3 and 4). Las Hoyas enantiornithines appear to have increased their PMR up to fivefold from youth to maturity (figures 2 and 5*b*). In our analysis, the hatchling metabolic scope ratio (PMR/RMR) is used as a proxy to infer developmental mode (figure 6). In altricial and semi-altricial hatchlings, which are highly dependent on parental care, RMR and PMR tend to be similar, resulting in a metabolic scope ratio approaching 1 [20]. By contrast, precocial and semi-precocial hatchlings are comparatively independent and capable of locomotion shortly after hatching. Their PMR exceeds the RMR to a greater extent, reflecting a broader aerobic scope [20]. In our sampled chick, the PMR is more than twice the RMR, indicating a metabolic scope between semi-precocial and precocial developmental strategies.

**Figure 6.**
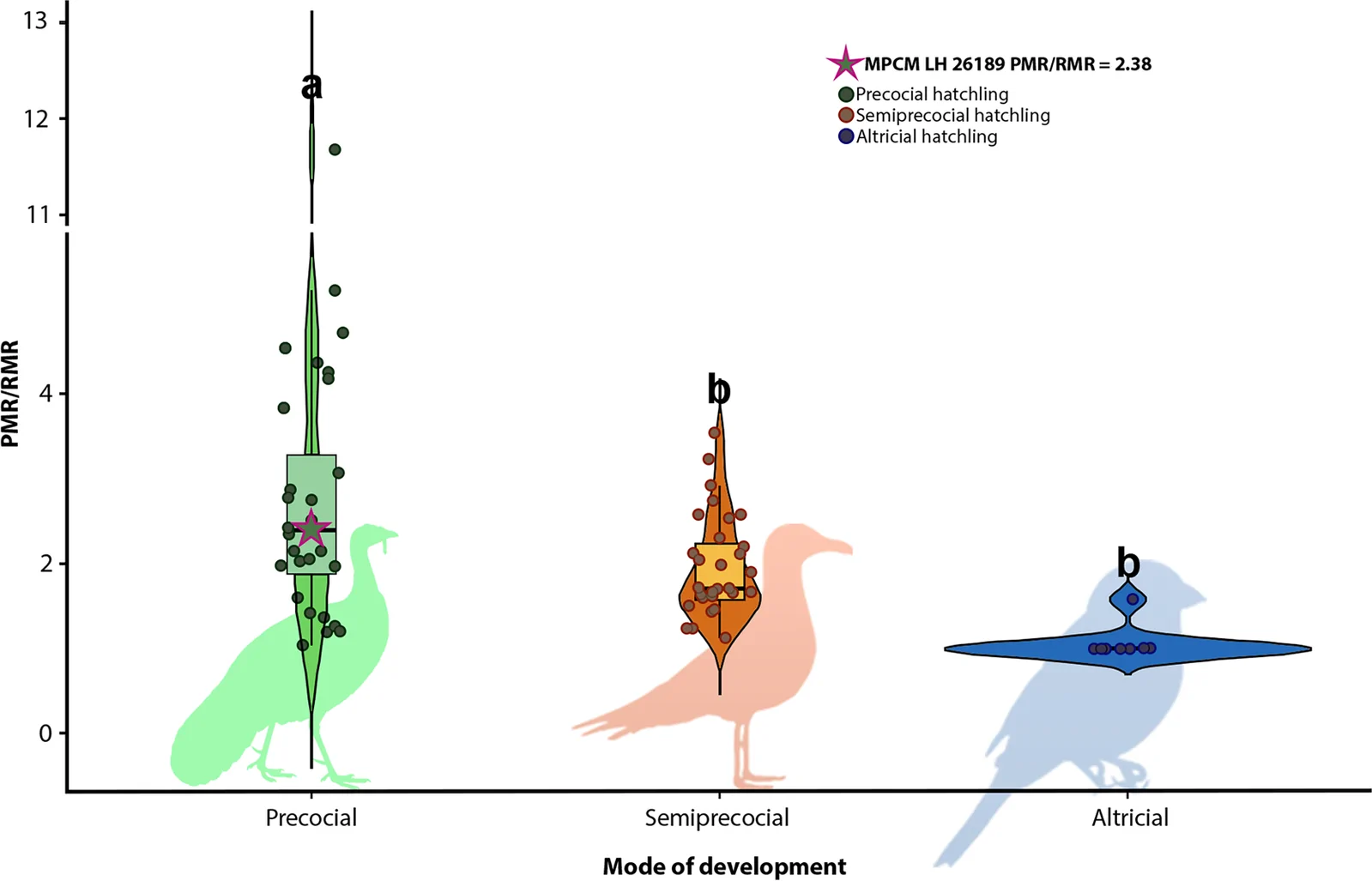
Distribution of metabolic scope (PMR/RMR) among extant hatchling birds with different developmental modes and retrodicted position of the enantiornithine MPCM-LH-26189. Violin plots show the distribution of PMR/RMR values for precocial (green), semi-precocial (orange) and altricial (blue) taxa. Box plots indicate the median and interquartile range, and data points are attributed to species values. The magenta-outlined star represents the PMR/RMR retrodicted for the hatchling MPCM-LH-26189 (PMR/RMR = 2.38). This fossil hatchling was classified as precocial. Letters above violins indicate significant differences among developmental modes based on linear model evaluation with randomized residuals in a permutation procedure pairwise comparisons; groups sharing different letters are significantly distinct. The outlier plotted corresponds to the duckling of *A. crecca*, which exhibits a mass-specific PMR of 8.08 mlO_2_ h^–1^g^–1^ approximately 12-fold higher than its mass-specific RMR of 0.69 mlO_2_ h^–1^g^–1^. The bird silhouettes have been modified and adapted from copyright-free images provided by Phylopic (http://www.phylopic.org).

Our predictive model classified the enantiornithine MPCM-LH-26189 as precocial, although its posterior probabilities were nearly equally split between precocial and semi-precocial developmental modes (figure 6). When integrated with osteological and histological evidence (e.g. [8,11,15,38,67]), these results support the hypothesis that enantiornithine hatchlings occupied a position along the semi-precocial/precocial spectrum, between which there is actually a thin divisory line [20]. Note that, given its small body size, MPCM-LH-26189 would have experienced substantial heat loss during periods of activity, as observed in extant precocial birds [73–75]. Thus, maintaining body temperature would have required a combination of shivering and parental brooding [20,75,76]. The metabolic scope of MPCM-LH-26189 suggests a capacity for thermogenesis and thermoregulation beyond that expected for altricial hatchlings [20], reflecting the development of skeletal musculature and supporting a degree of locomotor and physiological independence shortly after hatching.

### 4.3. Locomotor capacity of enantiornithine hatchlings

A distinctive enantiornithine trait is the presence of feathered wings in embryos and hatchlings of at least some species [8,68,77]. In extant birds, precocious species typically employ continuous flapping flight [78]. Several enantiornithines have been suggested as capable of this type of flight, including *Junornis* and *Gretcheniao* from Jehol and *Concornis* and *Eoalulavis* from Las Hoyas, and that they could alternate between continuous flapping and bounding flight to increase forward speed [9,79,80]. Nevertheless, the ability of enantiornithines to take wing shortly after hatching remains debated. MPCM-LH-26189 clearly had a largely cartilaginous sternum, insufficiently robust to support functional flight muscles (i.e. the *M. supracoracoideus* and *M. pectoralis*) and lacked well-developed feathers. We propose that this bird would have needed to reach a more advanced stage of ossification before flight was possible, with sufficient midline ossification to stiffen the sternum and enable it to withstand the increasing mechanical forces exerted by the flight musculature during ontogeny [8,66,81]. Before their sternum became ossified and sustained aerial flight was achieved, enantiornithine hatchlings may have probably been limited to climbing, using their wing claws in much the same way as hoatzin (*Opisthocomus hoazin*) chicks do; both taxa possess a weakly developed sternal keel [82–84]. With the sequential development and acquisition of the flight apparatus, a shift towards wing-assisted incline running (WAIR) may have occurred [85], in which partially developed wings aided their well-developed legs in ascending inclined surfaces during running. Prondvai *et al.* [86] found that hoatzin chicks exhibited an unusual degree of precocity in their wing and leg bones, a condition associated with their early ability to climb and swim. By contrast, ducklings exhibit precocial development of their leg bones, accompanied by delayed wing bone maturity, reflecting asynchronous skeletal development. The hatchling MPCM-LH-26189 possessed fully developed wing and leg bones, although delayed development of the sternum would have prevented it from achieving flight immediately after hatching. Its well-developed limbs may have enabled foraging soon after hatching, with powered flight becoming possible only after sufficient ossification of the sternum. Sternal ossification may have been stimulated by ground-based exercise of the wings before the chick's transition to the fledgling stage. Miller *et al.* [87] suggested that bohaiornithids occupied niches analogous to those of folivorous birds. Comparing the sternal ossification among enantiornithes, Zheng *et al.* [84] concluded that these birds retained basal sternal morphology characterized by a reduced keel throughout. An exception is represented by *Neuquenornis volans*, which exhibits a more pronounced carina.

Overall, young enantiornithines probably exhibited a precocial climbing, semi-precocial to precocial developmental mode, with a degree of post-hatching autonomy that included climbing, but without skeletal maturity required for immediate flight. As they matured, enantiornithines underwent a rapid increases in body size and improvement of physiological capabilities, reflecting rapid growth during early development. Atterholt *et al.* [66] suggested that growth strategies within enantiornithes were diverse and could include cyclical variation in growth rates and extensive remodelling. Our retrodictions support that metabolic capabilities in the sampled enantiornithines increased dramatically during early post-hatching ontogeny.

## 5. Conclusion

In conclusion, the enantiornithine hatchling MPCM-LH-26189 shows a metabolic scope falling within the semi-precocial to precocial spectrum of developmental strategies. While some studies proposed the possibility of flight soon after hatching in enantiornithines, owing to the presence of fledged individuals and well-ossified girdles in embryos and hatchlings [14,69,88], hatchlings that still exhibit incomplete sternal ossification, such as MPCM-LH-26189, show that early flight was unfeasible. Nevertheless, its elevated PMR suggests a capacity for thermoregulation and independence in foraging and climbing using the wing claws soon after hatching. Functional wing claws may have facilitated climbing trees prior to a developmental transition toward WAIR as an early stage of flight.

## 6. Future research directions

Several avenues of future research would further our understanding of avialan metabolic evolutionary implications through the method applied here. First and foremost, more altricial species should be sampled: one of the limitations of our study is the limited number of altricial species sampled for the analyses compared with the precocial taxa. A critical point in the current study is that hatchling long bones were not available for every taxon in the museum collections visited, so for many taxa we estimated the blood flow $\dot{Q}$ through the equation of Hu *et al.* [48]. Unfortunately, this regression equation is based on cursorial birds, which causes a limitation for the $\dot{Q}$ estimations in volant birds.

## Data Availability

All data including datasets, supplementary figures and R scripts for all analyses are archived on Zenodo https://doi.org/10.5281/zenodo.22058889 and available online [89].
